# epiGBS2: Improvements and evaluation of highly multiplexed, epiGBS‐based reduced representation bisulfite sequencing

**DOI:** 10.1111/1755-0998.13597

**Published:** 2022-03-03

**Authors:** Fleur Gawehns, Maarten Postuma, Morgane van Antro, Adam Nunn, Bernice Sepers, Samar Fatma, Thomas P. van Gurp, Niels C. A. M. Wagemaker, A. Christa Mateman, Slavica Milanovic‐Ivanovic, Ivo Groβe, Kees van Oers, Philippine Vergeer, Koen J. F. Verhoeven

**Affiliations:** ^1^ Bioinformatics Unit Netherlands Institute of Ecology (NIOO‐KNAW) Wageningen the Netherlands; ^2^ Department of Terrestrial Ecology Netherlands Institute of Ecology (NIOO‐KNAW) Wageningen the Netherlands; ^3^ Plant Ecology and Nature Conservation Group Wageningen University & Research (WUR) Wageningen the Netherlands; ^4^ ecSeq Bioinformatics GmbH Leipzig Germany; ^5^ Institut für Informatik Universität Leipzig Leipzig Germany; ^6^ Department of Animal Ecology Netherlands Institute of Ecology (NIOO‐KNAW) Wageningen the Netherlands; ^7^ Behavioural Ecology Group Wageningen University & Research (WUR) Wageningen the Netherlands; ^8^ Institute of Computer Science Martin Luther University Halle‐Wittenberg Halle Germany; ^9^ 6029 Plant Ecology and Physiology Radboud University Nijmegen the Netherlands; ^10^ 530625 German Centre for Integrative Biodiversity Research (iDiv) Halle‐Jena‐Leipzig Leipzig Germany

**Keywords:** bisulfite sequencing, *de novo* reference, DNA methylation, double digest, nonmodel species, reduced representation, SNP calling

## Abstract

Several reduced‐representation bisulfite sequencing methods have been developed in recent years to determine cytosine methylation *de novo* in nonmodel species. Here, we present epiGBS2, a laboratory protocol based on epiGBS with a revised and user‐friendly bioinformatics pipeline for a wide range of species with or without a reference genome. epiGBS2 is cost‐ and time‐efficient and the computational workflow is designed in a user‐friendly and reproducible manner. The library protocol allows a flexible choice of restriction enzymes and a double digest. The bioinformatics pipeline was integrated in the snakemake workflow management system, which makes the pipeline easy to execute and modular, and parameter settings for important computational steps flexible. We implemented bismark for alignment and methylation analysis and we preprocessed alignment files by double masking to enable single nucleotide polymorphism calling with freebayes (epifreebayes). The performance of several critical steps in epiGBS2 was evaluated against baseline data sets from *Arabidopsis thaliana* and great tit (*Parus major*), which confirmed its overall good performance. We provide a detailed description of the laboratory protocol and an extensive manual of the bioinformatics pipeline, which is publicly accessible on github (https://github.com/nioo‐knaw/epiGBS2) and zenodo (https://doi.org/10.5281/zenodo.4764652).

## 1 INTRODUCTION

Cytosine methylation at carbon position 5 (also termed 5‐meC) is a chemical epigenetic modification of DNA. This modification can influence gene activity and expression and has the potential to affect transcription regulation (Zhang et al., 2018). Genome‐wide 5‐meC discovery is routinely performed using methods based on bisulfite treatment followed by high‐throughput sequencing (BS‐Seq) (Reyna‐López et al., 1997). Whole genome BS‐Seq (WGBS) (Suzuki et al., 2018) is the gold standard if financial resources and a reference genome are available, which is still not the case for the majority of organisms. While the popularity of BS‐Seq studies is growing (Figure S1), data are mainly generated for model species such as mouse, human and *Arabidopsis thaliana*, representing 46%, 34% and 4% of all BS‐Seq data sets in the SRA (Table S1), respectively.

A less comprehensive but cheaper and versatile alternative to WGBS is BS‐Seq in reduced representations of the genome, by using restriction enzyme fragmentation during the library preparation, such as RRBS (Meissner et al., 2005), epiGBS (van Gurp et al., 2016), BsRADseq (Trucchi et al., 2016), epiRADseq (Schield et al., 2016) and Creepi (Werner et al., 2020). Several easy‐to‐use bioinformatics tools and workflows have been developed to analyse BS‐Seq data, such as BS‐Seeker2 (Guo et al., 2013), bismark (Krueger & Andrews, 2011) and bat (Kretzmer et al., 2017), which assume availability of a reference sequence for read mapping and methylation calling. However, there is increasing interest in studying DNA methylation in nonmodel study species, for instance to understand the involvement of DNA methylation in ecological and evolutionary processes. Such methods have to deal with the absence of reference genomes, the complex genomes of nonmodel organisms and high sample numbers, and have to accommodate a simultaneous comparison of genetic and epigenetic data, for instance to examine how much of the overall epigenetic variation between samples can be predicted from pairwise genetic relatedness (Richards et al., 2017).

In a previous publication, we presented epiGBS as a reduced‐representation DNA methylation analysis tool that combines those features (van Gurp et al., 2016). epiGBS calls both cytosine‐specific quantitative DNA methylation levels and single nucleotide polymorphisms (SNPs) from the same bisulfite‐converted samples, based on reconstructing the *de novo* consensus sequence of the targeted genomic loci. This means that the method can be applied also when no reference genome is available for the species under study (van Gurp et al., 2016). A similar approach was described by Werner et al. (2020), who provide a proof‐of‐concept via data obtained from almond and a *Pst*I single enzyme digest. Here, we present epiGBS2, which consists of a detailed, updated laboratory protocol and a revised computational analysis pipeline that is accessible for all with basic knowledge in bioinformatics, and we subject the method to several performance tests. Executing epiGBS2 is cost‐ and time‐efficient and is designed for user‐friendly, reproducible and flexible analysis, allowing for an effective determination of methylation and SNP variants in a broad range of species, including plants and vertebrates such as birds (Sepers et al., 2019). We evaluate the performance of epiGBS2 by comparing the analysis results of *A*. *thaliana* accessions and great tit (*Parus major*) samples to published benchmarking sets.

## 2 MATERIALS AND METHODS

### 2.1 Laboratory protocol

#### 2.1.1 Construction of epiGBS libraries

In order to reduce sequencing bias and costs, several major improvements were made to the original epiGBS laboratory protocol, the majority of which were described recently by Boquete et al. (2020). We briefly list the key improvements here. In addition, we present a detailed description of the adapter design, which allows flexible choice of the restriction enzyme pair, and a detailed step‐by‐step protocol of the library preparation in the Supporting Information. The resulting epiGBS2 library is paired‐end and directional, while additionally the information about the origin of the read is labelled by a control nucleotide. Reads originate either from the original top strand (Watson), the complementary Watson strand, the original bottom strand (Crick) or the complementary Crick strand.

#### 2.1.2 Identification of PCR duplicates

**FIGURE 1:**
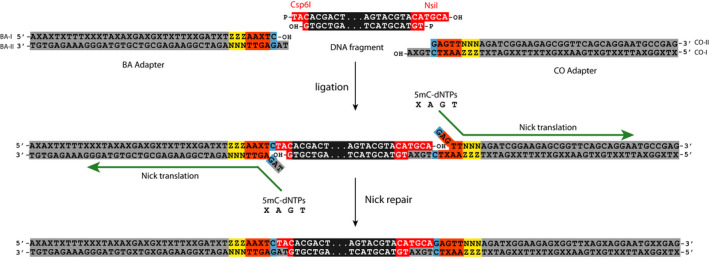
epiGBS2 uses hemimethylated adapters. epiGBS2 adapters consist of the Illumina adapter sequence (grey), a random 3 nucleotide sequence called UMI (yellow), a barcode (orange), a control nucleotide (blue) and the complement restriction enzyme site overhang sequence (grey). The 5′–3′ strand of the BA adapter and the 3′–5′ strand of the CO adapters contain only methylated cytosines (X); the opposite strands are unmethylated. All strands are dephosphorylated, so only adapter 3′ ends and DNA fragment 5′ ends ligate. During nick‐translation the “broken” strands are replaced with 5mC‐dNTPs, which results in fully methylated adapters

During the preparation of sequencing libraries, PCR clones can be produced. Removing these PCR duplicates computationally avoids overrepresented fragments caused by biased duplication, which allows for more accurate interpretation of results. Using common whole‐genome sequencing laboratory protocols, sequence identity is a basis for identifying PCR duplicates. However, in reduced‐representation approaches that use amplification of restriction enzyme‐associated DNA, fragments of identical sequence are produced by design; sequence identity is therefore not a basis for distinguishing PCR duplicates. To differentiate PCR duplicates from epiGBS sequencing reads that originate from different DNA molecules, a random three‐letter oligonucleotide was placed in the adapter sequence as described in van Moorsel et al. (2019) (Figure 1). This Unique Molecular Identifier (UMI) in combination with the read sequence is identical for PCR clones but different for reads that originate from different DNA molecules. This feature is used in the epiGBS2 computational workflow to specifically remove PCR clones.

#### 2.1.3 Use of a control nucleotide and a universal double restriction enzyme digest

**FIGURE 2:**
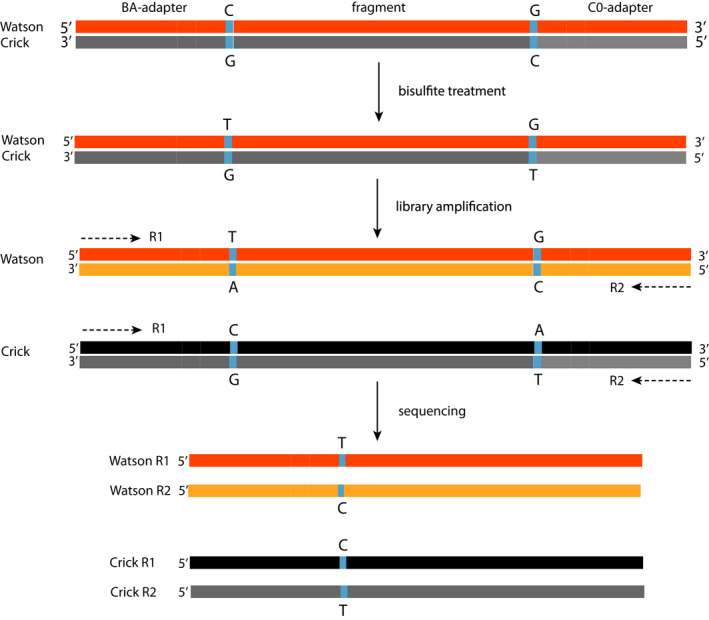
A control nucleotide (CN) is used for strand annotation. To allow a flexible choice in restriction enzymes but still be able to differentiate between original Watson (orange) and Crick (grey) strands, a control nucleotide (blue) was added to the epiGBS2 adapter, which consists of a single unmethylated cytosine in the BA‐I and the CO‐I adapters. During bisulfite treatment the cytosines are converted to thymines. After amplification the new bottom strand of the original Watson contains an adenine in the BA adapter but still a cytosine in the CO adapter; the new top strand of the original Crick contains a cytosine in the BA adapter and an adenine in the CO adapter. Subsequently, the R1 read of the Watson strand will contain a thymine at the control nucleotide position and a cytosine in the R2 read. R1 reads with a cytosine and a thymine in the R2 read will be annotated as Crick

The protocols of van Gurp et al. (2016) and Werner et al. (2020) used a single restriction enzyme (RE) with an unmethylated cytosine in the recognition site. To increase flexibility but keep the ability to differentiate Watson and Crick reads, we added the possibility to perform a double RE digest (e.g., with a rare and a frequent cutting RE) by introducing a “control nucleotide” (CN) in the adapter (see van Moorsel et al., 2019 for a further description). This CN is an unmethylated cytosine, which is placed after the barcode followed by the sequence of the RE overhang (Figure 1) and used for Watson/Crick annotation of the reads (Figure 2). Read pairs with T at the CN position of the R1‐/ adapter BA‐read and C at the CN position of the R2‐/ adapter CO‐read are defined as Watson; read pairs with C at the CN position of the R1‐/ adapter BA‐read and T at the CN position of the R2‐/ adapter CO‐read are defined as Crick. This design facilitates the use of various RE combinations and makes the epiGBS2 protocol more universally applicable. While the epiGBS protocol was originally optimized for plants, the freedom to also use other enzymes, such as for example *Msp*I, makes epiGBS2 now also very effective for studies on other organisms, such as vertebrates.

#### 2.1.4 Use of hemimethylated adapters

To reduce costs, the library preparation protocol was adjusted in such a way that hemimethylated adapter pairs are used instead of fully methylated adapters. In epiGBS2 the cytosines of the oligonucleotides adapter BA‐I and adapter CO‐I are 5‐C methylated (Figure 1, and see laboratory protocol in the Supporting Information). The oligonucleotides of the opposite strands (adapter BA‐II and adapter CO‐II) contain unmethylated cytosines only and are 5′‐dephosphorylated. After annealing the respective BA‐I and BA‐II and CO‐I and CO‐II adapter oligonucleotides and ligating them with the enzyme‐digested DNA fragment, only adapter 3′ ends and fragment 5′ ends ligate. A nick remains between adapter 5′ ends and fragment 3′ ends. The nick is repaired by using dNTPs that contain 5‐meC’s and that directly translate all 5′–3′ nucleotides starting from the nick. This results in fully methylated adapters that are ligated to the digested DNA fragment and a complementary 3‐nucleotide short UMI sequence.

#### 2.1.5 Miscellaneous optimizations

As with reduced‐representation sequencing (e.g., genotyping‐by‐sequencing) of unconverted DNA, the optimal adapter concentration can vary depending on the frequency of enzyme cut sites in the DNA and the genome size (Wallace & Mitchell, 2017). We therefore advise to ascertain the optimal adapter concentration for each new study system and restriction enzyme or enzyme combination by methods as suggested by Wallace and Mitchell (2017).

### 2.2 Computational analysis protocol

#### 2.2.1 Creation of a snakemake workflow

**FIGURE 3:**
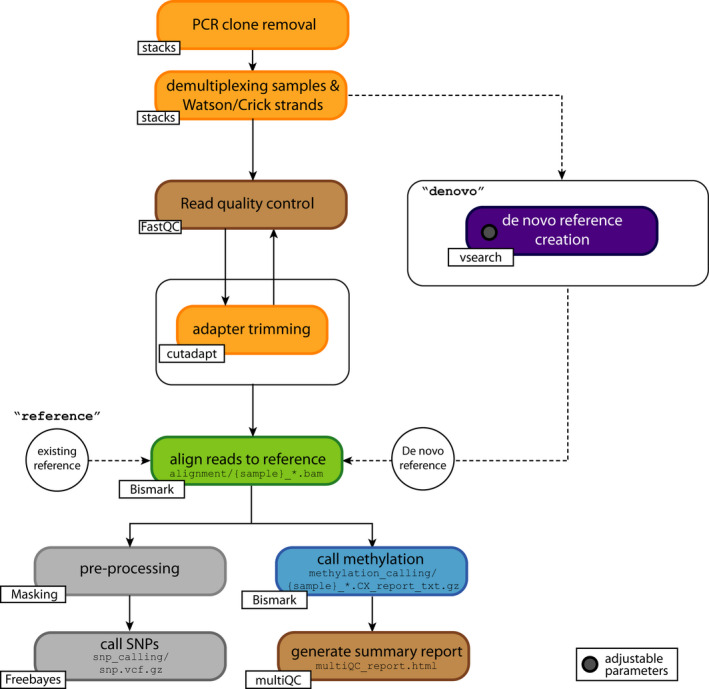
Overview of the epiGBS2 pipeline. The main snakemake rules are visualized. Boxes represent steps with modular output that can be executed individually. Orange: read preprocessing. PCR clones are removed from all input reads, and samples are demultiplexed with stacks2 and annotated as either Watson or Crick reads. These reads are adapter‐trimmed with cutadapt. Purple: in the *de novo* branch reads are either assembled with pear or joined with a custom script. These sequences are deduplicated, and Watson and Crick reads are paired and clustered based on identity. The minimum cluster size during deduplication and the identity percentage are introduced as variable parameters and can be set in the config file. Green: trimmed reads are aligned to the reference (either *de novo* clusters or pre‐existing reference) with bismark. Blue: methylation is also called with bismark. Grey: alignment files are preprocessed by double masking to enable SNP calling with freebayes. Brown: the processed reads (trimmed and untrimmed) are analysed in a read quality control using fastqc and summarized with the log files of all other crucial steps in a multiqc summary report

Workflow management systems (WMS), such as nextflow (Di Tommaso et al., 2017) or snakemake (Köster & Rahmann, 2012), are a way of describing analytical pipelines and computational tools. These systems have a common aim: to make computational methods reproducible, portable, maintainable and shareable. WMS assure monitoring of the progress of, for example, bash or python scripts and exit gracefully if any step fails (Perkel, 2019). WMS also integrate with package managers such as conda (Grüning et al., 2018) and docker (Merkel, 2014), which install software dependencies automatically, and allow flexible integration with resource management systems such as slurm. In combination, WMS and package managers make the installation and execution of analysis pipelines accessible for biologists who have a basic knowledge in bioinformatics. The main computational steps of epiGBS2 are PCR clone removal, demultiplexing and Watson/Crick strand annotation, read quality control, adapter trimming, mapping to a reference (*de novo* or pre‐existing), methylation calling and SNP calling. We embedded these steps in a snakemake (version 6.1.1) workflow (Figure 3) and divided them into specific rules that make the workflow modular and allow executing, exchanging or updating specific parts of the pipeline without modifying the others.

To simplify software installation and updating, we created conda (Grüning et al., 2018) environments in such a way that the workflow is portable and independent from the used Linux system. Each snakemake rule calls specific conda environment files and automatically installs the required dependencies. Custom scripts were written in Python 3.

#### 2.2.2 Description of the snakemake branches

epiGBS2 runs in two modes, which the user can select from the snakemake configuration file: either with a pre‐existing reference genome or in *de novo* mode. These two branches are nearly identical in all snakemake rules, except that in *de novo* mode the reference of the fragments under study is reconstructed from the epiGBS2 reads themselves (van Gurp et al., 2016) (Figure S2). The reference mode was added to the workflow to facilitate analysis in species for which a reference genome is available. Optionally, epiGBS2 can also be run in a third “legacy” mode. This mode runs a version of epiGBS2 that was described in Gawehns et al. (2020) and that reflects gradual updates to the epiGBS protocol since it was first published in 2016 (van Gurp et al., 2016); however, it differs substantially from the current and recommended *de novo* and reference branches. A detailed description of the computational tools, scripts and performance of the legacy branch can be found in the Supporting Information, which may be a useful reference for previous studies that used epiGBS analysis.

#### 2.2.3 Demultiplexing and Watson/Crick annotation

epiGBS2 takes raw sequencing reads and a barcode file as input. First, PCR clones are removed, which are identified by identical fragment and UMI sequence, using *clone_filter* from the stacks2 (Catchen et al., 2011, 2013, Rochette et al., 2019) software. Then a new barcode file is created automatically with a custom script, where each barcode combination is extended by the expected Watson (T in R1 and C in R2) and by the Crick control nucleotides (C in R1, T in R2), respectively. Reads are demultiplexed by sample and strand type (Watson or Crick) without allowing any mismatches in their barcode or control nucleotide sequence, using *process_radtags* from the stacks2 software. Only reads with confirmed presence of the expected RE overhang are retained. To correct for possible C/T conversions in the RE overhang sequence, one nucleotide mismatch is allowed. Such a mismatch is replaced with the expected nucleotide by using the ‐‐recover flag in stacks2. Using a custom script, read headers are labelled with their sample identification code and with either “Watson'’ or “Crick” for their usage in the *de novo* reference construction.

#### 2.2.4 *De novo* reference creation

*De novo* reference creation is performed as described in van Gurp et al. (2016) and in Figure S2. To implement this in the *de novo* branch of the snakemake workflow, the Watson and Crick R1 and R2 read files, which were created by merging the untrimmed reads of all samples after demultiplexing, are used as input. During R1‐ and R2‐read assembly with pear, 3′ end adapter sequences are removed. To reconstruct the consensus reference sequence, three clustering steps are performed: (i) deduplication of three‐letter encoded Watson and Crick reads; (ii) pairing of binary Watson and Crick reads; and (iii) clustering of reconstructed reference clusters by identity. Finally, the created *de novo* reference is prepared for the alignment with bismark by adding four N’s at the beginning and end of the clusters to ensure that bismark is able to align the reads to the *de novo* reference.

To allow adjustment of the *de novo* reference creation, we added the possibility to vary two *de novo* creation parameters directly from the snakemake configuration (Figure S2): The performance of the first deduplication step can be customized by setting a minimum and maximum cluster depth, and the last clustering step can be customized by setting the identity percentage for clustering. Fine‐tuning these parameters may optimize *de novo* reference construction depending on species‐specific genome characteristics.

#### 2.2.5 Adapter trimming

**FIGURE 4:**
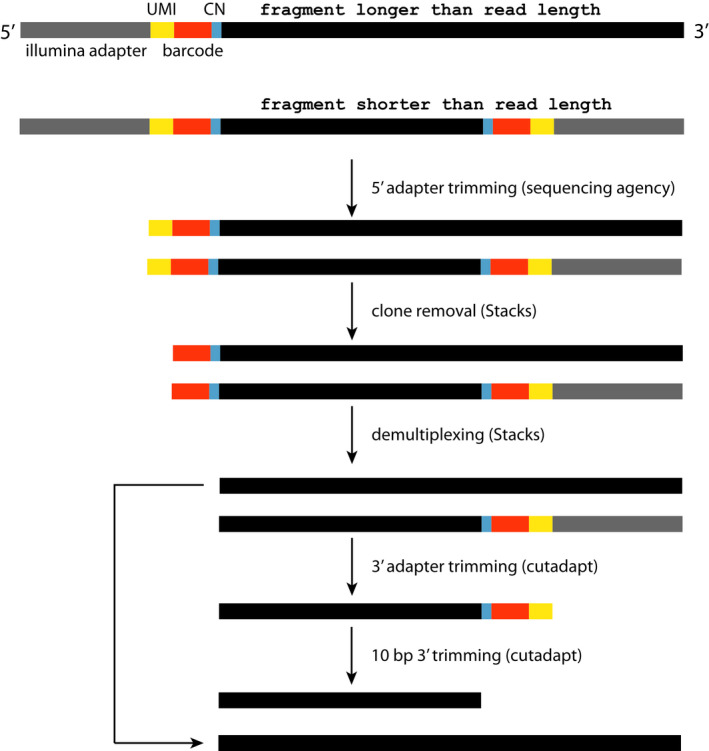
epiGBS2 reads need 3′ adapter trimming. When epiGBS2 fragments are shorter than the read length, sequencing continues into the adapter of the opposite fragment end. This 3′ adapter sequence has to be removed to ensure optimal performance during mapping, and SNP and methylation calling. Raw sequencing reads are usually 5′ adapter trimmed by the sequencing agency. During clone removal the UMI (yellow) sequence is removed, followed by trimming of the barcode (orange) and control nucleotide (blue) after demultiplexing. To trim the 3′ adapter sequence cutadapt is used; first the standard Illumina sequence is removed. Then additional 10 bp are hard trimmed, to discard also the custom part (UMI, barcode and control nucleotide) of the adapter sequence

To prepare the demultiplexed and strand‐annotated reads for the alignment step, they have to be adapter‐trimmed at the 3′ end, because the majority of the deduplicated and demultiplexed paired‐end reads are longer than the DNA fragment length. Therefore, reads are extended into the adapter sequence at the 3′ end of the fragment (Figure 4). Demultiplexed and Watson/Crick annotated read files are used as input per sample. To remove the standard Illumina adapters from the 3′ end, cutadapt (Martin, 2011) is used in paired‐end mode, which by default recognizes the commonly used Illumina adapters. Additionally, the first base of all reads is removed to avoid the use of filled‐in cytosines in the methylation calling and reads are filtered for a minimal length of 20 bp. Untrimmed reads are collected in a separate file and the read filtering step is repeated. To remove the custom part of the adapters (UMI, barcode, control nucleotide), the first 10 bp of the adapter trimmed reads are removed and filtered based on length (cut‐off 20 bp). Adapter‐trimmed and ‐untrimmed reads are merged, and then the combined Watson R1 reads are combined with the Crick R2 reads and the Watson R2 reads with the Crick R1 reads to create a conventional, directional library as it is expected by bismark and the SNP calling approach.

#### 2.2.6 Read mapping and methylation calling

Read mapping to the *de novo* clusters (*de novo* branch) or to a pre‐existing reference genome (reference branch), and subsequent DNA methylation calling, is implemented with bismark, a widely accepted tool for DNA methylation analysis. bismark’s output format can be used by different downstream analysis tools such as methylkit (Akalin et al., 2012) and dss (Park & Wu, 2016) and the tool itself is well maintained. Earlier implementations of methylation calling in epiGBS made use of samtools mpileup and custom scripts (see legacy branch; Supporting Information), which lacks the above‐named advantages.

After constructing of the genome index, alignment is performed in paired‐end mode using the merged read files that were created after trimming. bismark is executed with the following additional parameters:

○ save ‐‐un(aligned) and ‐‐ambiguous reads in separate files
○ ‐‐rg_tag: Write out a Read Group (RG) tag to the resulting SAM/BAM file
○ ‐‐rg_id: Sets the ID field in the @RG header line
○ ‐‐rg_sample: Sets the SM field (=sample name) in the @RG header line

From the alignment files, methylation is called by bismark using default parameters but with the following settings:

○ ‐p: Paired
○ ‐‐CX: Cytosine_report every single cytosine that was covered in the experiment irrespective of its sequence context
○ ‐‐no_overlap: for paired‐end reads it is possible that Read 1 and Read 2 overlap. This option avoids scoring overlapping methylation calls twice.
○ ‐‐scaffolds (in *de novo* mode only): Does not presort methylation calls into individual chromosome files. Instead, all input files are temporarily merged into a single file (unless there is only a single file), and this file is then sorted by both chromosome and position using the Unix sort command.

#### 2.2.7 Implementation of SNP calling

For SNP calling, we implemented a recently published approach (Nunn et al., 2021) that involves preprocessing of the bismark alignments using a custom script, followed by variant calling using freebayes (epifreebayes). This approach was shown to lead to increased precision and sensitivity of SNP calls compared to currently available bisulfite‐specific SNP calling methods (Nunn et al., 2021). epifreebayes exploits information from the opposite strand at any potentially converted or unconverted cytosine position when calling SNPs. First, all per‐sample alignment files are indexed and merged to a single combined alignment file using samtools (Li et al., 2009) (version 1.11). After indexing this file, the MD tag, a string encoding mismatched and deleted reference bases, is added using samtools calmd. The indexed output is preprocessed with a double‐masking procedure, ensuring that variant calling is not affected by the bisulfite conversion, as explained in Nunn et al. (2021) and variants are called with freebayes using the following parameters:

○ ‐‐no‐partial‐observations: Causes freebayes to only consider observations that completely cover the haplotype window that is used during calling
○ ‐‐report‐genotype‐likelihood‐max: Report genotypes using the maximum‐likelihood estimate provided from genotype likelihoods
○ ‐‐genotype‐qualities: Output genotype qualities (GQ) for filtering
○ ‐‐min‐coverage 0: Require at least this coverage to process a site. Default 0
○ ‐‐min‐base‐quality 1: Exclude alleles from analysis if their supporting base quality is less than 1. Default 20
○ ‐‐min‐mapping‐quality 10: Exclude alleles from analysis if their supporting mapping quality is less than 10. Default: 30
○ ‐‐no‐population‐priors: Assume that samples result from pooled sequencing and turns off Ewen's Sampling Formula component of priors

#### 2.2.8 Creation of a summary output file

To improve user‐friendliness, the pipeline reports the log files of all critical steps in a single combined file, created by multiqc (Ewels et al., 2016) (version 1.8). Custom multiqc modules and a custom parsing script (src/report/parse‐logs.py) were used for software log files that are not recognized by default. The report consists of the following parts:

○ Clone Removal Statistics: Shows the number and the percentage of clone reads that are removed during PCR clone removal using the UMI sequence information.
○ fastqc (Andrews, 2010) (version 0.11.4) statistics for untrimmed and trimmed, demultiplexed and Watson/Crick‐annotated reads.
○ Demultiplexing statistics: Shows ambiguous barcode drops (reads without any of the expected barcode combinations), low‐quality read drops (reads with a raw phred score below 10, based on a sliding window approach), ambiguous RAD‐Tag drops (reads without the expected RE overhang, allowing for one nucleotide mismatch), retained reads for all samples (total number of demultiplexed reads).
○ *De novo* Barcodes: During demultiplexing the software also recognizes barcode combinations at the 5′ end of the sequencing reads that are not in the barcode file provided by the user. Barcode combinations with a high number of reads and with both the Watson and the Crick control nucleotide combination should be investigated in more detail. Note that the program might also recognize parts of the restriction overhang as part of the barcode sequence.
○ Read Assembly statistics (*de novo* mode only): pear assembly statistics about merging R1 and R2 reads for Watson and Crick strands individually.
○ *De novo* Identity Clustering (*de novo* mode only): Statistics of the last clustering step based on identity in the *de novo* consensus construction.
○ cutadapt: reports the 3′ end adapter removal.
○ bismark: reports the alignment statistics, cytosine methylation percentages and M‐bias per sample.

### 2.3 Validation experiments

#### 2.3.1 *Arabidopsis thaliana* samples and description of benchmarking data

*A*. *thaliana* individuals from six different accession (Col‐0, Gu‐0, Ler‐0, C24, Ei‐2 and Cvi‐0; six to eight individual plants per accession) were grown under standard glasshouse conditions and leaf tissue DNA was isolated from 5‐week‐old flowering plants using the Macherey‐Nagel NucleoSpin‐ Plant II kit (using PL1 lysis buffer). The epiGBS2 library preparation was conducted following the protocol described in the Supporting Information, resulting in a single, multiplexed sequencing library containing 44 barcoded samples (eight C24; eight Col‐0; eight Cvi‐0; seven Ei‐2; eight Gu‐0 and three Ler‐0). The library was sequenced at Novogene on a HiSeq X system (Illumina) and general read quality filtering and adapter trimming were executed by the company. The sequencing reads were analysed using the *de novo*, legacy and reference branches of the epiGBS2 pipeline. In the *de novo* and legacy branch, min‐depth =10, max‐depth =10,000 and clustering id =0.99 were used for the *de novo* reference creation. The *A*. *thaliana* TAIR_10 assembly was used as reference (GCA_000001735.1). To evaluate the performance of epiGBS2 methylation calling, the methylomes of the accessions available in the 1001 epigenomes project (Kawakatsu et al., 2016) were used as a benchmark. However, Ler‐0 methylome information was missing. Similarly, to evaluate the SNP calling performance of epiGBS2, obtained results were compared to SNPs obtained in the 1001 genomes project (1001 Genome Consortium, 2016).

#### 2.3.2 *Parus major* samples and description of benchmarking data

To analyse the performance of the epiGBS2 reference branch in a vertebrate species, four individual *P*. *major* samples from an epiGBS2 library were compared to reduced representation bisulfite sequencing (RRBS) data (Meissner et al., 2005) from the exact same samples. These four individuals were part of a former RRBS study (Sepers et al., 2021). For detailed methods on sample collection and DNA isolation see Sepers et al. (2021). The epiGBS2 library preparation was conducted following the laboratory protocol described in the Supporting Information, with the modification that genomic DNA was digested with the restriction enzymes *Msp*I and *Nsi*I. As the four samples were pooled with 44 other samples, this resulted in a single multiplexed sequencing library containing 48 barcoded samples which was sequenced on an Illumina HiSeq X (150 bp from paired‐end reads) by Novogene. Reads were demultiplexed using epiGBS2 allowing one mismatch in their R1 barcode and control nucleotide sequence and zero mismatches in their R2 barcode and control nucleotide sequence. Raw reads were checked for quality and adapter content using fastqc version 0.11.8 (Andrews, 2010). fastq screen version 0.11.1 (Wingett & Andrews, 2018) in bisulfite mode was used to detect possible contaminations with pre‐existing databases and indexed genomes: Phix (Coliphage phi‐X174), vectors (UniVec Core), *A*. *thaliana* (thale cress, TAIR_10), *Escherichia coli* (*E*. *coli* strain K‐12 substr. MG1655) and *Homo sapiens* (Genome Reference Consortium Human Build 38). Reads were merged before trimming by combining Watson R1 reads with the Crick R1 reads and the Watson R2 with the Crick R2 reads. Adapter trimming was executed as in the epiGBS2 reference branch, but additionally the first three bases of all R2 reads and the first 12 bp of the adapter‐trimmed R2 were removed. Quality improvement of the reads was verified by fastqc, fastq screen and multiqc. Trimmed reads were aligned to the *P*. *major* reference genome v1.1 (GCF_001522545.3) (Laine et al., 2016). Alignment was run in nondirectional mode and with the parameters ‐‐un(aligned) and ‐‐ambiguous only. Methylation was called in CpG context only and with the options ‐p, ‐‐no_overlap, ‐‐report, ‐‐bedGraph, ‐‐scaffolds and ‐‐cytosine_report.

RRBS libraries were prepared with an *Msp*I single digest. Construction of the libraries and sequencing (Illumina HiSeq 4000, 100 bp from single‐end reads, by Roy J. Carver Biotechnology Centre, University of Illinois at Urbana‐Champaign) was done as described in Sepers et al. (2021) but with the following changes: an Ovation RRBS Methyl‐seq System 1‐16 kit from NuGEN was used, where the fragments were amplified with PCR amplification (12 cycles) and the restriction fragments were size selected to a range of 20–200 bp, by amplified library purification with beads (Agencourt RNAClean XP Beads, two washes) to produce the final library. Data processing was performed as described in Sepers et al. (2021), except that diversity bases and reads without an *Msp*I signature (CGG or TGG) at the 5′ end were removed using a custom python script (https://github.com/nugentechnologies/NuMetRRBS/blob/master/trimRRBSdiversityAdaptCustomers.py). To remove the diversity bases, 0–3 bases were trimmed at the 5′ end and 5 bases were trimmed on the 3′ end. Furthermore, alignment was done using bismark version 0.22.3 (Krueger & Andrews, 2011) using default parameters. Subsequently, methylation calling in only CpG context was done using bismark with the options ‐s, ‐‐report, ‐‐bedGraph, ‐‐scaffolds, ‐‐cytosine_report and ‐‐genome_folder.

Filtering of epiGBS and RRBS methylation calls was done with R version 4.0.1 and the R package methylkit version 1.16.1 (Akalin et al., 2012). For each individual, sites that were not present in both data sets (RRBS and epiGBS2) and sites with low coverage (<10) were excluded. The destrand option was set to TRUE to combine C’s in the same CpG site (CpG dinucleotides). The performance of the epiGBS2 data was evaluated with correlation plots that were created in R (version 4.0.1) (R Core Team, 2021), using ggplot2 version 3.3.3 (Wickham, 2016). The coefficient of determination (*R* ^2^) was calculated using the ggpmisc package version 0.3.9 (Aphalo, 2021).

#### 2.3.3 Evaluation of epiGBS2 de novo reference creation

To analyse the performance of epiGBS2 *de novo* reference creation, the clusters generated by the epiGBS2 *de novo* branch analysis of *A*. *thaliana* samples were aligned to the TAIR_10 reference genome using minimap2 (2.17‐r941) (Li, 2018) with the alignment criteria as set in the genomic short read preset (‐x sr). The *de novo* reference clusters were generated based on pooled data from all six accessions used in the sequencing library. The mapping percentage was used to determine the quality of the *de novo* reference creation. To match the epiGBS2 *de novo* reference with the TAIR_10 reference, as necessary for subsequent comparison of cytosine methylation and SNP calls, the nucleotide positions of the *de novo* clusters were adjusted using a custom python script (src/lift_over.py).

#### 2.3.4 Performance testing of epiGBS2 methylation calling

When comparing methylation calls from the epiGBS2 study to available epigenomes in the 1001 epigenome project, effects of stochastic methylation differences between individuals were minimized by summarizing methylation and nonmethylation calls of individual samples as an average per accession. We compared methylation calls at cytosines that had a minimum coverage of 10× in the 1001 epigenome reference data and a minimum average coverage of 10× per individual sample in the epiGBS2 data (i.e., a total of 10× the number of individuals included for that accession). For the comparison between the epiGBS2 *de novo* and reference branches, methylation calls per sample were not averaged to generate an accession‐level estimate and a 10× coverage threshold per sample was applied. Correlation plots were created in R (version 4.0.1) (R Core Team, 2021), using ggplot2 (Wickham, 2016), and *R* ^2^ was calculated using the ggpmisc package (Aphalo, 2021).

#### 2.3.5 Performance testing of epiGBS2 SNP calling

To test the SNP calling performance, all variants that were not SNPs but, for example indels, were removed from the variant calling output (vcf files) of the reference branch. SNP positions of the 1001 genome project that were not covered by the epiGBS2 output were removed. Due to strand bias in RRBS methods during sequencing, and because potential cytosine positions are only called in epifreebayes when reads are also available from the opposite strand, a subset of true SNPs are undetectable by the epiGBS2 pipeline. To gain insight in the magnitude of this problem, we filtered the epiGBS2 and 1001 genome data by only keeping those positions for which at least one read of each strand (top and bottom) were present in the epiGBS2 data. The performance testing of SNP calling was done with rtg vcfeval (Cleary et al., 2015) (version 3.11) with variant quality (QUAL) as qualifier, to evaluate the sensitivity and precision of epiGBS2 SNP calls. Sensitivity is defined as the number of true positives divided by the number of SNPs in the baseline data set (in other words: How many of the true SNPs are detected by epiGBS2?) and precision is defined as the number of true positives divided by the total number of discovered variants (how many of the epiGBS2‐called SNPs are true SNPs?). Furthermore, the ‐‐squash‐ploidy parameter was used to account for the lack of heterozygous genotypes in the 1001 genomes data sets by ignoring the genotype information in the estimation of precision and sensitivity. The performance test was executed for each individual sample but only results from sample Cvi‐0_11 are shown here. Plots were created in R (R Core Team, 2021) using ggplot2 (Wickham, 2016).

## 3 RESULTS

### 3.1 Short description of execution of epiGBS2 pipeline

After executing a paired‐end next generation sequencing run, the sequencing reads should be 5′‐adapter trimmed as executed by most sequencing agencies, but custom parts (UMI, barcode, control nucleotide and restriction site overhang) should remain. The reads of individual samples are multiplexed, so two input files in fastq format will be received for the bioinformatics workflow: Read 1 (forward reads, usually indicated by “R1” in the file name) and Read 2 (reverse reads, usually indicated by “R2” in the file name). The following steps have to be taken to successfully run the workflow (for more details see documentation of the epiGBS2 code: https://github.com/nioo‐knaw/epiGBS2#readme):

1. Check technical hardware requirements.
2. Retrieve the epiGBS2 pipeline from github (https://github.com/nioo‐knaw/epiGBS2) or zenodo (https://doi.org/10.5281/zenodo.4764652).
3. Fill out the config file.
4. Prepare a barcode file.
5. Start the pipeline.
6. Check the status of the pipeline regularly for errors.
7. After the process has finished or an error occurred, inspect the snakemake output and the pipeline report.
8. Check output files as described in the documentation.

All predicted methylation sites are reported per sample in multiple output formats such as the cytosine report which describes the context, number of methylated and unmethylated calls, and methylation percentage per site. This format can be used as input in common downstream analysis packages such as methylkit (Akalin et al., 2012) or dss. The SNP calls are summarized in a single multicohort vcf file, which can be used to perform downstream genetic analysis, such as genetic map construction, population genomics or phylogenetics.

### 3.2 3′ End adapter removal

To evaluate the performance of 3′ end Illumina and custom adapter removal as described in the Material and Methods section, the read adapter content of sample Cvi‐0_11 was determined by fastqc. Directly after demultiplexing and strand annotation, up to 18% of reads contained Illumina adapter sequences at the 3′ end (Figure S3). After the additional trimming and filtering step with cutadapt, these sequences were removed. Consequently, average read size decreased from 140 to 130 bp for R1 reads and from 142 to 129 bp for R2 reads, but most reads (99.99%) were retained. The amount of reads with a high per‐sequence base quality increased, the percentage of overrepresented sequences decreased, and no increase of cytosines (R1 reads) and guanines (R2 reads) was observed at the end of the reads after the trimming was performed.

### 3.3 Creating *de novo* reference sequences from the epiGBS2 reads

To investigate the performance of the epiGBS2 *de novo* reference creation, the epiGBS2‐generated reference clusters were mapped against the *A. thaliana* reference genome using minimap2 (Li, 2018). In total, 87.69% of the 19,766 *de novo* clusters uniquely aligned to the reference; 10,960 of these clusters aligned without any mismatches, and the mean number of mismatches between the TAIR_10 genome and the *de novo* reference was 1.1, indicating that the sequences of most of them were reconstructed successfully. It also demonstrates that the large majority of *de novo* clusters do not derive from contaminating DNA, such as endosymbionts. When mapping demultiplexed and trimmed epiGBS2 reads to the TAIR_10 genome using the reference branch, mapping percentages ranged between 50% and 70% depending on accession, with Col‐0 performing best. The *de novo* branch performed similarly, with mapping percentages between 48% and 57%. This again indicates overall good performance of the *de novo* reference construction.

**FIGURE 5:**
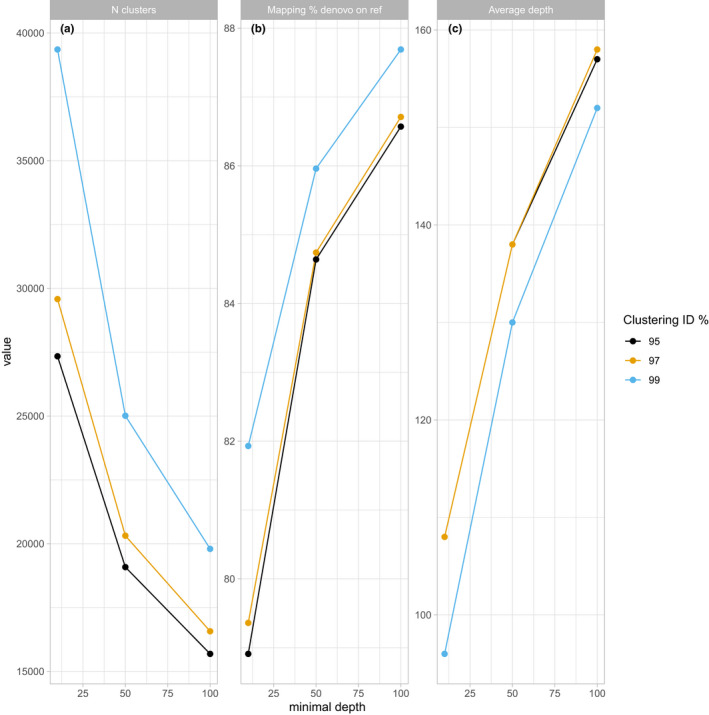
Effects of clustering parameter settings on *de novo* reference creation of *Arabidopsis. thaliana*. To evaluate the influence of clustering parameters on the *de novo* reference, the effects of minimal cluster depth of the first clustering step (*x*‐axis: 10, 50, 100) and the last clustering step (cluster identity: black 95%, orange 97%, blue 99%) on the number of created *de novo* reference clusters (a), the percentage of read mapping against the *de novo* reference (b) and the average read depth per site after mapping (c) were determined

Next, we investigated the effect of the *de novo* cluster creation parameters (minimal depth of the first clustering step and clustering identity) on critical quality values of the clustering steps. When increasing the minimal cluster depth, the number of final *de novo* clusters decreases, while by increasing the identity percentage more clusters are created (Figure 5a). At high minimal cluster depth, and hence lower cluster numbers, the mapping percentage against the *de novo* reference increased (Figure 5b) because more reads could be mapped uniquely (Figure S4). At lower minimal cluster depth, higher clustering identities result in higher mapping percentages; however, when the minimal cluster depth is increased, higher clustering identity does not lead to higher mapping percentage. These clustering parameter settings, by affecting the percentage of uniquely mapped reads, also affect average sequencing coverage per site, which is highest when the minimal cluster depth is higher and the identity percentage is lower (Figure 5c). Coverage, in turn, influences the methylation calls (Figure S4). In conclusion, and similar to other *de novo* reference‐based methods, we recommend exploration of the *de novo* reference creation parameters when working with new species to ensure optimal performance (Paris et al., 2017).

### 3.4 epiGBS2 methylation calling

**FIGURE 6:**
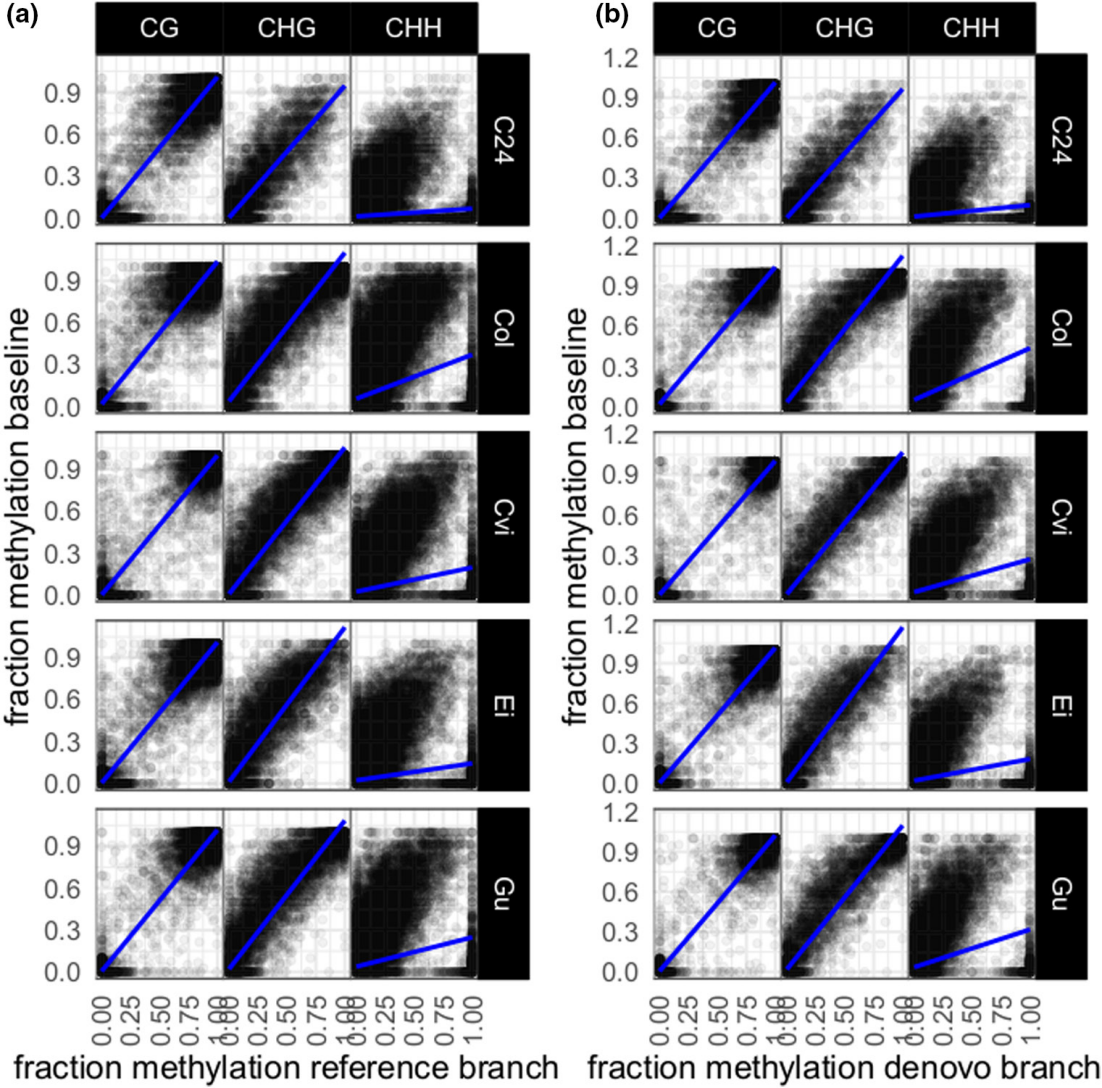
Methylation calls of the epiGBS2 reference and *de novo* branches correlate with baseline data for cytosine methylation in CG and CHG contexts. Methylation was called for the pooled individuals of five different *Arabidopsis. thaliana* accessions (C24, Col‐0, Cvi‐0, Ei‐2, Gu‐0) in the epiGBS2 reference branch (a) and *de novo* branch (b). Scatter plots were created to compare those calls with the methylation calls of the baseline data set in three different cytosine contexts (CG, CHG, CHH)

*A. thaliana* cytosine methylation was called from reference and *de novo* branch alignments. In total, 1,047,597 and 1,827,467 sites were obtained in the *de novo* and reference branch, respectively, of which 928,070 sites overlapped. The methylation calls of all sites were plotted against the calls of the 1001 epigenome project (baseline data). For both branches and all tested accessions, the epiGBS2 methylation calls in CG context were strongly correlated with the baseline methylation calls (*R* ^2^ > .93) (Figure 6). In CHG context, the correlation was slightly lower (reference branch *R* ^2^ > .79, *de novo* branch *R* ^2^ > .8). For CHH context, very low correlations were found between the epiGBS2 calls and the baseline data set. Methylation in CG context is known to show high levels of transgenerational stability (Graaf et al., 2015), and therefore high similarities in CG methylation between individuals from the same *A*. *thaliana* accession are expected, even when plants are grown in different environments and have unknown generational distances within the accession. The observed high correlation between the epiGBS2 samples and the 1001 epigenome benchmarking data shows that epiGBS2 methylation calling performs well. CHH methylation, on the other hand, is much more variable among conditions (Dubin et al., 2015) and hence a much lower correlation is expected, which may reflect biological difference rather than technical differences of the methylation calling procedure. Methylation calling of the legacy branch produced near‐identical results to the epiGBS2 *de novo* and reference branches in all three methylation contexts (Figure S5).

### 3.5 epiGBS2 SNP calling

**FIGURE 7:**
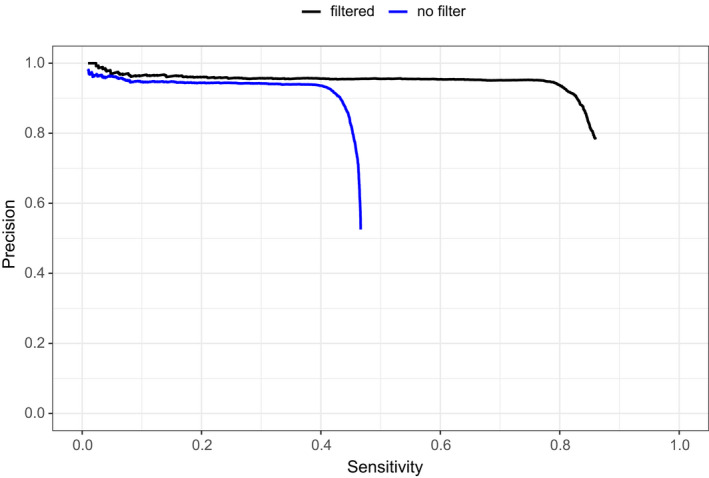
Precision of SNP calling in epiGBS2 is high. SNPs were called for a single *Arabidopsis. thaliana* Cvi‐0 individual (Cvi0_11) with the epiGBS2 reference branch using the TAIR_10 reference genome. The SNP calling was evaluated against the Cvi‐0 baseline data to assess precision and sensitivity. Precision–sensitivity plots were created with Rtg vcfeval and show precision and sensitivity estimates for a continuous range of different SNP qualities. Results from filtered data consider all positions present in the baseline data that are covered by epiGBS2 reads on both strands (top and bottom strand). In contrast, all baseline positions are considered in the unfiltered data. Because epiGBS2 can only call SNPs involving a potentially converted or unconverted cytosine when both strands are covered, a higher proportion of baseline SNPs remain undetected by epiGBS2 in the unfiltered data, resulting in reduced sensitivity

To evaluate the SNP calling, a comparison of filtered and unfiltered SNP calls between the epiGBS2 analysis and the baseline set was performed with Rtg vcfeval. The precision and sensitivity of epiGBS2 SNP calling were determined as a function of variant quality (QUAL), which is calculated by the variant caller (freebayes) and a phred‐based metric describing the probability that a certain position contains a variant. Selecting for SNPs with higher quality scores is expected to lead to higher precision (more of the epiGBS2 SNP calls are correct) but lower sensitivity (because more sites, and thus also more true SNPs, are excluded from evaluation because they do not meet the quality filter threshold). At high SNP quality thresholds, more than 90% of the SNPs identified in the epiGBS2 reference branch corresponded with the baseline data set (Figure 7). At lower quality values a larger total of true baseline SNPs were detected by epiGBS2, but this also resulted in an increased false positive rate (for instance, only half of the called SNPs are true positives at a quality value of 10; Figure 7). Because RRBS methods are prone to strand bias but SNPs in cytosine context can only be called using opposite‐strand alignment, a subset of true baseline SNPs remain undetectable and limit the level of sensitivity that can be maximally achieved in the unfiltered data. When we excluded sites that were not covered at both strands from the epiGBS2‐baseline comparison (the filtered data set), sensitivity increased: ~80% of the remaining SNPs in the baseline were detected by epiGBS2 (Figure 7). Because heterozygous genotypes were not called in the baseline set (1001 Genome Consortium, 2016), we ignored the genotype calls during the SNP benchmarking by treating the genomes as haploid. Including the evaluation of correct genotype calling in the benchmarking, in addition to the correct allele, the overall level of precision was reduced in both filtered and unfiltered subsets (Figure S6). Further investigation revealed this difference to be driven mainly by a fraction of true homozygous SNPs that were misidentified as heterozygous under epiGBS2. A similar excess of heterozygous SNP calls from WGS and simulated WGBS data from *A*. *thaliana* Cvi‐0, compared to the 1001 genomes benchmark data, was observed in Nunn et al. (2021) when using freebayes. This suggests that the freebayes tool, rather than inferring SNPs from bisulfite data, is causing the small discrepancy with the benchmark data.

**FIGURE 8:**
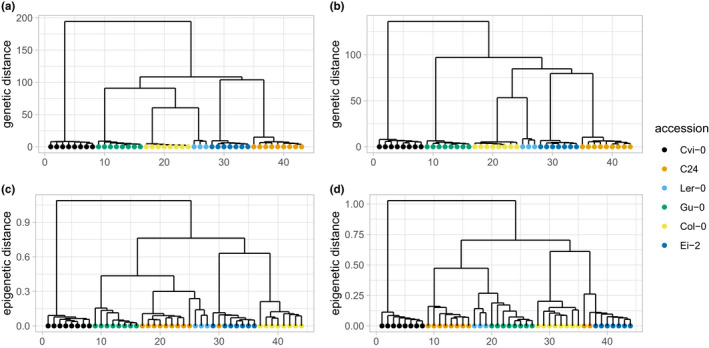
*Arabidopsis thaliana* accessions are differentiated based on epiGBS2 methylation or SNP calls. SNPs (a, b) and methylation (c, d) were called for six different *A*. *thaliana* accessions by the epiGBS reference branch (a, c) or *de novo* branch (b, d). Genetic relatedness between accessions was calculated using SNP calls and using Euclidian distance. Clustering was performed using the Ward method. Distance calculation of methylation calls was executed using clusterSamples() in methylkit (Akalin et al., 2012) based on correlation distances and clustered using the Ward method

To illustrate that epiGBS2 produces DNA methylation and SNP calls that differentiate the used *A*. *thaliana* accessions, we determined the relatedness between the samples. This was performed by calculating correlation and Euclidean distances for methylation and SNP calls, respectively, followed by clustering using the Ward method. Based on methylation data (Figure 8a,b) and on SNP calls (Figure 8c,d), samples clustered by accession both in the *de novo* and reference branch analyses. Only two C24 samples did not cluster correctly with other samples from the same accession based on methylation calls. The number of reads assigned to one of these samples was lower than from other samples, which might influence reliable methylation calling, and also led to exclusion of this sample from the SNP analysis. The number of reads of the other sample was comparable to the overall average; hence, the observed difference might be biological or has an unknown technical reason.

### 3.6 Performance epiGBS vs. RRBS

**FIGURE 9:**
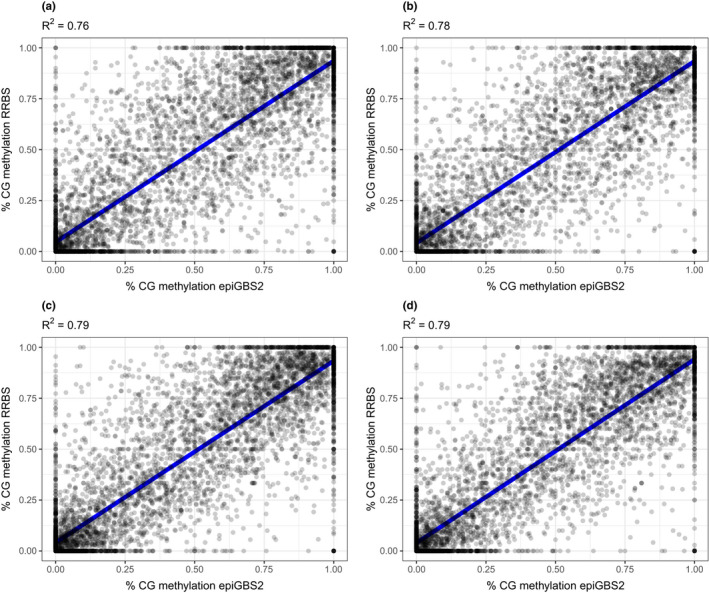
DNA methylation calls from epiGBS2 and RRBS are correlated. Comparison of DNA methylation calls from epiGBS2 and RRBS data sets. CG methylation was called from epiGBS2 and RRBS data from four *Parus major* individuals (a–d) and scatterplots were created to determine correlation between the two methods

In the *P. major* data, before filtering, the number of unique CpG sites in an epiGBS2 sample ranged from 189,960 to 199,933, with 130,543 unique CpG sites with a mean coverage of 24.56 in the whole epiGBS2 data set. The number of unique CpG sites in an RRBS sample ranged from 2,516,396 to 2,579,571, with 2,267,295 unique CpG sites with a mean coverage of 27.77 in the whole RRBS data set. For each *P*. *major* individual, 5832–6969 CpG sites were shared between RRBS and epiGBS2 after filtering. CpG methylation was called from the epiGBS2 samples, plotted against the calls of the RRBS data and the correlation coefficient determined. For all individuals, the epiGBS2 methylation calls correlated with the RRBS‐derived methylation calls (*R* ^2^ ≥ .76) (Figure 9). Hence, due to the use of different REs, largely different parts of the genome were sampled, but within the existing overlap, methylation levels were very similar.

## 4 DISCUSSION

We presented here epiGBS2, consisting of a detailed description of our current laboratory protocol and a bioinformatics workflow for epiGBS analysis. epiGBS2 includes several major updates compared to the method as first published by van Gurp et al. (2016), a performance test and a user‐friendly computational analysis pipeline aimed at the researcher with some basic experience in bioinformatics, such as working in a Linux environment. The updates were made in part to improve the method but also to make the method available in a more user‐friendly and reproducible way to users working on a wide range of organisms. To date, published papers using epiGBS have been mainly limited to plant species (e.g. Alvarez et al., 2020; Moorsel et al., 2019; Mouginot et al., 2021; Mounger et al., 2021; Prudencio et al., 2018), but also include studies from vertebrates (Meröndun et al., 2019) and molluscs (Johnson & Kelly, 2020). By making the use of restriction enzymes more flexible (including double RE digest), epiGBS2 allows for easier implementation in many different organisms, including those where standard RRBS used to be the preferred method. Our validation results show that critical steps of the method perform well, resulting in accurate DNA methylation calls and highly precise SNP calling.

### 4.1 Validation of epiGBS2 results

Evaluation of the epiGBS2 pipeline performance was done by performing epiGBS2 analysis in several *Arabidopsis thaliana* benchmarking accessions, for which detailed SNP and DNA methylation information was available from previous whole‐genome sequencing and BS‐Seq studies. Comparing epiGBS2 results to these baseline data provided strong support that cytosine methylation levels are identified correctly in both CG and CHG contexts, in both the *de novo* and reference branches of epiGBS2. In CHH context, however, epiGBS2‐based DNA methylation results did not correlate well with the benchmarking data. In plant genomes, DNA methylation in CHH context is known to be much more dynamic and environment‐dependent than methylation in other cytosine contexts (Dubin et al., 2015). Thus, we interpret the lack of a good correlation in CHH context as probably reflecting real differences in CHH methylation between the samples that were used in our study and the samples that were used in the 1001 epigenomes project, our baseline data. This could be caused, for instance, by differences in growing conditions between the studies.

Both the reference and the *de novo* branches were able to correctly differentiate the *A*. *thaliana* accessions under study based on epiGBS2 SNP calls. The reference branch showed high SNP calling precision, indicating that few false positives were identified. SNP calling sensitivity is low due to the inherent strand bias of epiGBS2. However, when considering only positions filtered for coverage on both strands, epiGBS2‐based SNP calling performed equally well to SNP calling based on WGBS data from the same *A*. *thaliana* Cvi‐0 accession, as benchmarked previously in a different study (Nunn et al., 2021). Precision‐sensitivity of SNPs was not directly evaluated on data from the *de novo* branch because no comparable truth set exists, as the reference sequences are themselves derived during the execution of the pipeline. Relative to the TAIR 10 genome, for example, reference and alternative alleles can ostensibly switch places depending on their prevalence throughout the selected population of accessions for *A*. *thaliana*. The variant calling procedure is otherwise identical between the two branches, however, and the relative performance on the *de novo* branch can be indirectly inferred from (i) the level of precision‐sensitivity on the reference branch, in combination with (ii) the high rate of mapping *de novo* sequences, and (iii) the distance‐based clustering of accessions showing the expected relatedness when derived from SNP variants obtained with the *de novo* branch.

Validation of epiGBS2 results was also conducted by comparing epiGBS2 and RRBS data from four great tit samples. Despite a relatively low number of shared sites for this specific comparison, comparing epiGBS2 to the RRBS data supports that cytosine methylation levels in CG context are correctly identified. We expect the number of shared sites and the correlation to be even higher if the same restriction enzymes were used and if the computational analysis protocol had been identical. However, the use of an *Msp*I restriction digest without *Nsi*I as usually done in RRBS has to be evaluated in future studies, as we did not investigate the performance of epiGBS2 for enzymes with symmetric cut‐sites. Nonetheless, the observed correlation between the *P. major* epiGBS2 samples and the RRBS data shows that epiGBS2 methylation calling performs well in a vertebrate species.

### 4.2 Limitations and future work

Making epiGBS2 available allows others to use the updated methodology. However, we note that the development of epiGBS2 is a continuous process. Some known but minor shortcomings of the bioinformatics procedures are as follows. For instance, during demultiplexing the presence of the expected RE overhangs is validated. This validation accepts one nucleotide mismatch to allow recognition of C‐to‐T converted RE overhang sequences after bisulfite treatment. If a mismatching nucleotide is identified (e.g., a T instead of a C), it is replaced with a C by the stacks2 code; this can effectively result in an unmethylated cytosine becoming labelled as methylated. One possible solution could be for a script to approve the remaining RE overhang and allow C/T conversions without replacing them.

Another known issue is that we often find that 20%–30% of all sequencing reads cannot be demultiplexed successfully due to ambiguous barcodes. We identified two possible contributors to this issue: (i) in all epiGBS2 data sets that we inspected, the *de novo* barcodes recognized by stacks2 contain a notable number of sequences that resemble existing barcode combinations but that miss the first nucleotide of the R1 barcode. When inspecting untrimmed reads with the UMI still attached, we observed that the missing barcode nucleotide is typically present but only two UMI nucleotides are identified. This indicates that the issue may be caused by the UMI sequences. UMI sequences were synthesized at random, so it is possible that some low‐complexity UMIs are generated that prevent optimal phasing of the sequencing. (ii) In epiGBS2 data sets with high PCR duplication rates, we identified *de novo* barcode combinations with an existing R2 barcode sequence, which we also found in the R1. However, in this combination, these specific barcode pairs were not expected. A similar issue was noted by Trucchi et al. (2016) in a related protocol, which was attributed to improper annealing during PCR of the forward primer to the CO adapter and which may be remedied by modifying the primer design (Trucchi et al., 2016).

Regarding the laboratory protocol, a recent change to the epiGBS2 adapter and primer design involves the incorporation of an Illumina index in the fragments during library preparation (see laboratory protocol in the Supporting Information). All epiGBS2 data presented here were produced on Illumina Hiseq lanes, where a full lane was dedicated to a single library, and no Illumina index was included in the fragments. With the recent move to higher‐volume sequencing platforms (e.g., Illumina Novaseq), addition of an Illumina index may be necessary to facilitate identification of the epiGBS2 reads by the sequencing facility, when epiGBS2 libraries are pooled with libraries of other customers in a single sequencing lane. We describe barcode design that includes an Illumina index in the library protocol (see Supporting Information). Further evaluation of epiGBS2 data generated on the Novaseq platform is desirable and currently ongoing.

Another improvement could be made in achieving uniformity in sequencing output per sample. The epiGBS2 protocol prioritizes efficiency and cost‐effectiveness in library preparation; however, in our hands, we observe considerable between‐sample variability in read counts after sequencing. This might be improved by conducting size selection of fragments after RE digest for each individual sample before adapter ligation. Alternatively, improvement can be achieved by quantifying DNA amounts per individual sample with a qPCR step after adapter ligation. This makes the library preparation process more elaborate, but may provide more control over individual sample output. Alternatively, the sequencing performance of individual barcodes and barcode combinations could be benchmarked in more detail.

## 5 CONCLUSION

We have here presented and validated epiGBS2, an updated method and analysis pipeline of previously published epiGBS approaches and showed that it performs well in terms of methylation and SNP calling compared to “gold standards” such as WGBS. The detailed description of the adapter design and the use of a CN allows for flexible use of different restriction enzymes and makes epiGBS2 applicable to a wide range of organisms and applications. However, before starting with new organisms and RE combinations we highly recommend to (i) estimate the expected complexity reduction by an *in silico* digestion of the genome of the species of interest or a related species, (ii) optimize the adapter concentrations as described in Wallace and Mitchell (2017) and (iii) run the *de novo* reference clustering with different parameter settings as those will influence the mappability of the sequencing reads. Embedding the analysis pipeline in snakemake makes the execution of the pipeline more accessible to nonbioinformaticians, more user‐friendly and reproducible, and allows the user to exchange the tools for mapping, methylation and SNP calling to software of their own choice.

## AUTHOR CONTRIBUTIONS

Koen J. F. Verhoeven, Fleur Gawehns, Philippine Vergeer and Kees van Oers designed the research; Morgane van Antro, Bernice Sepers, A. Christa Mateman and Slavica Milanovic‐Ivanovic performed the experiments; Thomas P. van Gurp, Niels C. A. M. Wagemaker, Adam Nunn and Samar Fatma contributed to improvements to the protocol, new reagents and analytical tools; Fleur Gawehns, Maarten Postuma and Bernice Sepers analysed data; Koen J. F. Verhoeven, Maarten Postuma and Fleur Gawehns wrote the paper with input from all coauthors.

## CONFLICT OF INTEREST

The authors have no conflict of interest to declare.

### OPEN RESEARCH BADGES

This article has earned an Open Data Badge for making publicly available the digitally‐shareable data necessary to reproduce the reported results. The bioinformatics pipeline and documentation can be accessed on github (https://github.com/nioo‐knaw/epiGBS2) and was deposited on zenodo (https://doi.org/10.5281/zenodo.4764652).

## Supporting information

Supplementary Material

## Data Availability

The demultiplexed *A*. *thaliana* epiGBS data were deposited on NCBI with BioProject ID PRJNA764918 and raw data files are accessible on Zenodo (https://doi.org/10.5281/zenodo.5519370). Great tit data can be accessed on NCBI with BioProject ID PRJNA208335. The RRBS reads are available under the SRA accessions SRX10989860, SRX10989861, SRX10989862 and SRX10989863, and the epiGBS2 reads are available under the SRA accessions SRX10994457, SRX10994458, SRX10994459 and SRX10994460. The epiGBS2 laboratory protocol can be found in the Supporting Information. The bioinformatics pipeline and documentation can be accessed on github (https://github.com/nioo‐knaw/epiGBS2) and was deposited on Zenodo (https://doi.org/10.5281/zenodo.4764652). An example data set can also be accessed on Zenodo (https://zenodo.org/record/5878925). All scripts used for benchmarking are available at https://github.com/MaartenPostuma/epiGBS‐Benchmarking.
